# A knot quicker and easier than Whip stitching in anterior cruciate ligament reconstruction

**DOI:** 10.1308/003588412X13373405385214m

**Published:** 2012-07

**Authors:** E Leong, M Lemon

**Affiliations:** Royal Surrey County Hospital nHS foundation Trust,UK

We describe a method for tying a self-locking knot to apply tension to a free tendon end for hamstring graft anterior cruciate ligament reconstruction. This is faster, safer and easier than whip stitching and is secure enough to feed the graft through bone tunnels.

**Figure 1:**
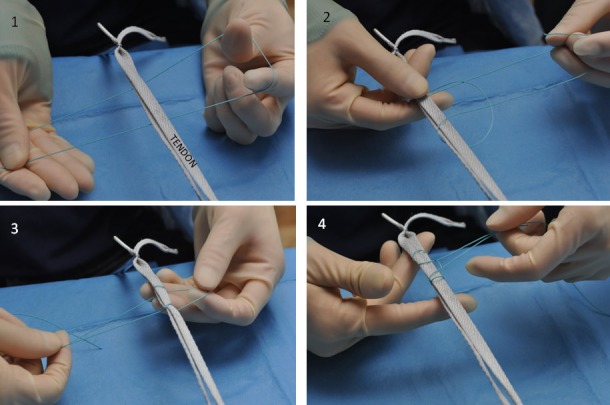
Method for tying a self-locking knot

The suture is folded and the tendon is laid on top (Fig 1a). The suture ends are then fed over the tendon and through the loop (Fig 1b). This is repeated (Fig 1c). The end result is shown in Figure 1d. The knot is pulled tight and a square knot is tied around the tendon to secure it.

